# A Comprehensive Study of the Microclimate-Induced Conservation Risks in Hypogeal Sites: The *Mithraeum of the Baths of Caracalla* (Rome)

**DOI:** 10.3390/s20113310

**Published:** 2020-06-10

**Authors:** Francesca Frasca, Elena Verticchio, Alessia Caratelli, Chiara Bertolin, Dario Camuffo, Anna Maria Siani

**Affiliations:** 1 Department of Physics, Sapienza Università di Roma, P.le A. Moro 5, 00185 Rome, Italy; annamaria.siani@uniroma1.it; 2 Department of Earth Sciences, Sapienza Università di Roma, P.le A. Moro 5, 00185 Rome, Italy; elena.verticchio@uniroma1.it; 3 Tecno.EL S.r.l., Electronic Technologies Company, Via degli Olmetti 38, 00060 Formello, Italy; a.caratelli@tecno-el.it; 4 Department of Mechanical and Industrial Engineering, Norwegian University of Science and Technology, 7491 Trondheim, Norway; chiara.bertolin@ntnu.no; 5 National Research Council (CNR), Institute of Atmospheric Sciences and Climate (ISAC), Corso Stati Uniti 4, 35127 Padua, Italy; d.camuffo@isac.cnr.it

**Keywords:** risk index, conservation assessment, microclimate, efflorescence, phase diagram, bio-colonisation, cultural hypogeal sites, mithraeum

## Abstract

The peculiar microclimate inside cultural hypogeal sites needs to be carefully investigated. This study presents a methodology that aimed at providing a user-friendly assessment of the frequently occurring hazards in such sites. A Risk Index was specifically defined as the percentage of time for which the hygrothermal values lie in ranges that are considered to be hazardous for conservation. An environmental monitoring campaign that was conducted over the past ten years inside the Mithraeum of the Baths of Caracalla (Rome) allowed for us to study the deterioration before and after a maintenance intervention. The general microclimate assessment and the specific conservation risk assessment were both carried out. The former made it possible to investigate the influence of the outdoor weather conditions on the indoor climate and estimate condensation and evaporation responsible for salts crystallisation/dissolution and bio-colonisation. The latter took hygrothermal conditions that were close to wall surfaces to analyse the data distribution on diagrams with critical curves of deliquescence salts, mould germination, and growth. The intervention mitigated the risk of efflorescence thanks to reduced evaporation, while promoting the risk of bioproliferation due to increased condensation. The Risk Index provided a quantitative measure of the individual risks and their synergism towards a more comprehensive understanding of the microclimate-induced risks.

## 1. Introduction

In recent decades, microclimate analysis has played a key role in the field of preventive conservation, as it allows researchers to investigate which environmental conditions are responsible for the deterioration that was observed on movable and immovable cultural objects. Indeed, understanding the interactions between artwork and surrounding environment means finding solutions for appropriate preventive conservation.

Cultural and natural hypogeal sites constitute a unique and valuable part of world heritage. A list of subterranean UNESCO sites—grouped by cultural, natural, and mixed heritage—is provided by Trofimova and Trofimov [1]. The term hypogeum refers to different types of subterranean sites: from natural (e.g., caves) to man-made sites (e.g., catacombs, tombs, crypts, foundations, etc.), built above ground and covered by soil or dug into the rocks. Hypogeal sites are characterised by stable temperature and high relative humidity throughout the year. On a yearly basis the indoor mean temperature reflects the outdoor one, whereas the outdoor daily thermal cycle extinguishes a few decimetres below ground level and the seasonal thermal cycle at around 10 m below ground level. These features are affected by the combined effect of the homogenous heat capacity of the envelope and limited indoor-outdoor air exchanges [2]. Other features of hypogeal sites are low or absent natural light and the abundance of organic and inorganic nutrients as well as soluble salts, which could be transported by percolating water from seepage and soil or by visitors. The stable environmental conditions characterising hypogeal sites are usually not harmful for preservation, but it is fundamental to be aware that this fragile equilibrium could be broken by sudden fresh air infiltrations or exposure to natural/artificial light.

In the last twenty years, several studies have investigated the indoor climate of natural and/or cultural hypogeal sites. A list of recent publications on this topic is given in Table 1. These works focus on the analysis of measured and/or simulated hygrothermal variables, sometimes along with other environmental data (e.g., irradiance, oxygen, or carbon dioxide concentrations). Some of these studies focus on identifying and preventing the causes of deterioration, e.g., biological and/or crystallisation risks, while others consider the effect of visitors on the sites or, in the case of hypogeal settlements, their energy performance. Nevertheless, none of the listed papers provide a thorough and straightforward approach for the characterisation of microclimate-induced conservation risks.

The crystallisation of salts is one of the frequently occurring factors of the deterioration of porous materials in hypogeal sites [7,8,9]. It is visible at the macro-scale as efflorescences and sub-efflorescences progressing towards the loss of material from superficial to inner layers in terms of powdering, detachments, blistering, exfoliation, and flaking. Crystallisation is influenced by the evaporation of saline solutions, which strongly promotes crystal growth from large to small pores, thereby inducing stresses in the material micro-structure. Several mechanisms are involved, such as crystallisation pressure, thermal expansion, osmotic pressure, and chemical weathering. Common deliquescent salts are sulfates, chlorides, and nitrates, which can often be found in association with cement that is used for restoration interventions [15], percolation of rainwater from the surrounding soils [16,17] and wet or deposition of airborne particles on surfaces [18]. Price at al. [19] and López-Arce et al. [18] proposed strategies to control rising damp and, hence dissolution, mobilisation, and crystallisation of salts. Nevertheless, the natural ventilation of the site should be limited in order to reduce moisture evaporation from walls governing dissolution/precipitation cycles.

The proliferation of biodeteriogens is another detrimental factor for preservation within hypogeal sites [3,4,5]. The presence of biodeteriogens is promoted by the availability of liquid water on surfaces in conjunction with nutrients, natural/artificial light and poor indoor ventilation. Biodeteriogens can form a complex mucous matrix, in which they are embedded, called *biofilm*. Biofilms contribute remarkably to rock and building material decay and, depending on the type of biodeteriogen, they usually appear as coloured patinas, which may exert a detrimental aesthetic impact. In Roman hypogeal sites, the main agents that are responsible for biodeterioration are cyanobacteria, i.e., photosynthetic microbes pioneering biofilms by forming a resistant extracellular polymeric substrate. Once the biofilms are formed, heterotrophic biodeteriogen, such as actinobacteria and fungi, can colonise the surface by exploiting the metabolites and biomass that are produced by cyanobacteria [20,21]. Bruno et al. [22] employed an LED lighting system to limit the growth of biofilm by inhibiting photosynthetic activity. Natural ventilation of the site should be favoured in order to reduce spore concentration in the air as well as the moisture evaporation from walls.

This paper presents a comprehensive study that is devoted to the assessment of the microclimate-induced conservation risks inside hypogeal sites. The methodology is applied to the “Mithraeum of the Baths of Caracalla”, a Roman hypogeal site, which suffered from different decay processes and where a long-term microclimate monitoring campaign is still in operation. The case study and the research aims are described in the following paragraphs.

### 1.1. The Case Study

The “Mithraeum of the Baths of Caracalla” (hereafter called Mithraeum) is one of the most important sites of Mithraism in Rome, as it preserves a valuable fresco of Mithras in the Central Hall. It was built in the early 3rd century AD in an underground corridor of the north-west *exedra* of the Roman Baths. The Mithraeum, at a depth of 10–15 m, covers a total area of 600 m${}^{2}$ and has five communicating rooms, where the Central Hall was the main room of the Mithraism cult. The site was built in *opus testaceum* made of two external parallel terracotta bricks (*bipedus spezzati*) filled with a layer of lime mortar and inhomogeneous aggregate (earth, tuff, and smashed bricks) for a wall total thickness of 1.5 m.

Several deterioration processes were observed in 2010 in the Central Hall: exfoliation, flaking, and swelling due to salt weathering and green/dark patinas caused by biological colonisation. A microclimate monitoring campaign was started in December 2010 to assess the effect of microclimate on deterioration processes and, thus, to seek solutions for improving site conservation. A maintenance intervention was carried out in 2011 to reduce internal ventilation with the aim of inhibiting efflorescence through the closure of the main openings, i.e., the open lateral windows (*“bocche di lupo”*) and the roof covering of an adjoining room. The adopted measure visibly reduced the occurrences of efflorescence and sub-efflorescence due to salts crystallisation, but did not inhibit the biological proliferation. Since then, the microclimate monitoring has been ongoing, although not consecutively, over all months of each year, due to a lack of funds and technical issues.

### 1.2. Research Aims

The aim of this research is to outline a comprehensive approach for the assessment of the microclimate-induced deterioration in cultural hypogeal sites through the definition of a Risk Index. The Risk Index (RI) is targeted at allowing a fast and reliable evaluation of the risk of crystallisation/dissolution of salts and bio-colonisation. The availability of the long-term microclimate data collected in the “Mithraeum of the Baths of Caracalla” provides the opportunity to test the ability of the RI to show the synergism of frequently occurring hazards inside hypogeal sites.

## 2. Materials and Methods

### 2.1. Microclimate Monitoring Campaign

**Figure 1:**
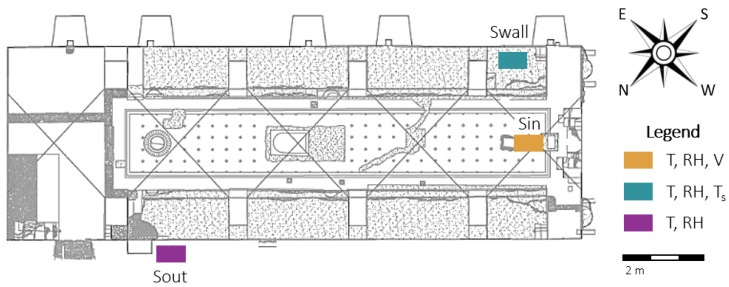
Position of the sensors in the Central Hall of the Mithraeum.

Four configurations of the microclimate monitoring system were tested in the first place. No significant differences were found among the temperature (T) and relative humidity (RH) values that were collected in the different positions [23]. This first investigation allowed for fine-tuning the deployment of sensors, as depicted in Figure 1.

The monitoring system consists of one thermo-hygrometer placed outside the Mithraeum at 1.8 m above the ground (S${}_{out}$), one thermo-hygrometer together with a biaxial sonic anemometer placed in the Central Hall (S${}_{in}$), and on thermo-hygrometer together with a surface temperature (T${}_{s}$) sensor above ground fixed close to a south-east-facing wall (S${}_{wall}$). The biaxial anemometer (distributed by Gill Instruments—Wind Sonic) was installed to measure the air flow intensity (V) and direction. The thermo-hygrometers (distributed by Rotronic HygroClip^®^ HC2-S(3)), commonly used for indoor climate monitoring, were chosen in accordance with European Standards EN 15758:2010 [24] and EN 16242:2012 [25]. However, at high RH levels (above 90%), the dielectric material of the capacitive sensor can be saturated by water vapour, which implies a significant error in the RH measurement together with an uncertainty increase equal to ±6% [26]. Thus, in 2015, the thermo-hygrometers were replaced by a new generation of instruments (distributed by Rotronic HygroClip^®^ HC2-S(3)-HEATED) that use heated capacitive sensors to avoid saturation of the dielectric material [14]. All of the sensors were connected to the GRILLO MMTS data logger for data transmission to a cloud server for real-time visualisation and remote download. The sampling interval was set to 5 min. and the processing time to 30 min., providing the minimum, maximum, and the average of the recorded variables.

### 2.2. Microclimate Data Analysis

The long time series of microclimate data that were collected over the years were objectively evaluated with the Completeness Index (CoI) and the Continuity Index (CI), defined in [27], in order to assess the quality of the datasets before applying data analysis.

Mixing Ratio (MR) and Dew Point temperature (T${}_{d}$) were calculated from the recorded T and RH data using the *formulae* provided in EN 16242:2012 [25]. Both of the variables describe a conservative property of the air parcel: MR provides the total amount of vapour water [28], whereas T${}_{d}$ pinpoints isobaric cooling without the addition or subtraction of water vapour [29]. The higher the RH, the lesser the cooling that should be experienced to reach saturation. Another conservative reference for the moisture content of moist air is the wet bulb temperature (T${}_{w}$), which is the temperature that is to be reached through an isobaric cooling due to the evaporation of water. If the wall surface temperature T${}_{s}$ is lower than T${}_{d}$, condensation occurs on the surface. The higher the RH, the smaller the amount of water that should be evaporated to reach saturation.

For diagnostic purposes, T${}_{s}$ values can be used to determine whether a surface is condensing or evaporating: T${}_{d}$ is the temperature related to condensation while T${}_{w}$ is the temperature related to evaporation. If T${}_{s}$ < T${}_{d}$, moisture is condensing; if T${}_{s}$ = T${}_{w}$ or T${}_{s}$ > T${}_{d}$, the wall is evaporating. The theory is simple, but the parameters that are involved can be obtained from indoor measurements with the help of empirical formulae, which, by their very nature, are built in statistical terms. However, a logical connection between variables is possible, and will be explained in the following formulae.

The temperature difference ΔT${}_{d}$ = T${}_{s}$− T${}_{d}$ allows for an evaluation of the condensation on the wall surface: when $\Delta T_{d}>0$, there is no condensation, when $\Delta T_{d}<0$, the larger the distance of T${}_{s}$ from T${}_{d}$, the greater the condensation.

The temperature difference ΔT${}_{w}$ = T${}_{s}$− T${}_{w}$ allows for evaluation of the evaporation from the wall surfaces: when T${}_{s}$ > T${}_{w}$, the evaporation occurs. The larger the distance of T${}_{s}$ from T${}_{w}$, the greater the evaporation. The ΔT${}_{w}$ value can be calculated after the readings of air temperature T and ΔT${}_{d}$ while using the empirical formula given in [29]:

(1) $$\Delta T_{w}=T_{s}-T_{w}=\Delta T_{d}\cdot \left(a_{1}\cdot \Delta T_{d}+a_{2}\cdot T^{2}+a_{3}\cdot T+a_{4}\right)$$

where the coefficients have the following values: a${}_{1}$ = −8.89 × 10${}^{-3}$ °C${}^{-1}$; a${}_{2}$ = −1.28 × 10${}^{-4}$ ${}^{{}^{\circ}}$C${}^{-2}$; a${}_{3}$ = 1.79 × 10${}^{-2}$ ${}^{{}^{\circ}}$C${}^{-1}$; and, a${}_{4}$ = $3.68\times 10^{-1}$.

As the most frequent conservation risks in hypogeal sites are related to evaporation and condensation, two empirical functions were calculated in order to study both the evaporation rate E${}_{aero}$ (mm · day${}^{-1}$) and the condensed water CW (mg · cm${}^{-2}$) in proximity of walls. E${}_{aero}$ was calculated according to the simplified Penman equation [30] while taking into account the maximum daily temperature T${}_{max}$ (${}^{{}^{\circ}}$C), the average daily relative humidity RH (%), and the average air flow intensity V (m · s${}^{-1}$):

(2) $$E_{aero}=b_{1}\cdot \left(T_{max}+b_{2}\right)\cdot \left(\frac{100-\mathrm{RH}}{100}\right)\cdot \left(1+b_{3}\cdot V\right)$$

where the coefficients are: b${}_{1}$ = 0.049 mm·day${}^{-1}$·${}^{{}^{\circ}}$C${}^{-1}$; b${}_{2}$ = 16.3 ${}^{{}^{\circ}}$C; b${}_{3}$ = 0.536 m${}^{-1}$. This formula establishes that evaporation is strongly favoured by high air temperature, low RH, and ventilation intensity.

The amount of water that condenses on walls, CW, has been calculated with an experimentally derived formula that links the amount of water vapour dissolved in 1 kg of dry air (MR), the difference between the dew point and the surface temperature (T${}_{d}$− T${}_{s}$), and the length of the period P during which T${}_{s}$ < T${}_{d}$, normalised in days of permanence below the dew point [31]:

(3) $$\mathrm{CW}=c_{w}\cdot \mathrm{MR}\cdot \left(T_{d}{-}_{s}\right)\cdot P$$

This formula establishes that the amount of water that condenses on walls is proportional to the moisture content in air (MR), the spread between the dew point and the wall temperature ($T_{d}-$T${}_{s}$), and the time duration P in which the wall temperature is lower than the dew point. The proportionality coefficient c${}_{w}$ depends on the intensity of the local ventilation, expressed in terms of air flow intensity V, i.e.,:

(4) $$c_{w}=c_{1}+c_{2}\cdot V$$

where the coefficients c${}_{1}$ = 1.194 and c${}_{2}$ = 0.140 m${}^{-1}\cdot$ s. The higher the air flow intensity, the faster the condensation rate. The coefficient c${}_{1}$ represents the condensation rate when the ambient air is still, and it is due to the diffusivity of water vapour in still air; c${}_{2}$ is related to the transport of moisture by advection.

### 2.3. Conservation Risk Assessment

In hypogeal sites, crystallisation and biological risks predominantly affect the conservation of wall surfaces, which may sometimes be decorated with by frescoes, such as in the Mithreum. For this reason, surface temperature (T${}_{s}$) and relative humidity at the surface–air interface (RH${}_{s}$) were used to evaluate the conservation risk in proximity of walls. RH${}_{s}$ values were estimated using the inverse formula in [25] and assuming the MR gradient from ambient air in proximity to wall surface as constant.

To assess the principal chemical-physical risks, assumptions were based on typical occurrences of deliquescent salts in Roman sites [16]. Chlorides, nitrates, and sulfates are frequently found in tuffaceous/volcanic material and in pozzolana-made structures that are typical of Roman sites [3]. Sulfates and nitrates are also common when the structure is in direct contact with the ground, as the most soluble compounds dissolved within soils percolate through seepages of rainwater or irrigation water [16]. Even chlorides can be found in the percolating water if the site is affected by marine aerosol or in the case of chloride-bearing geological formations. Our work analysed separate salt systems, i.e., chlorides, nitrates, and sulfates, which, however, could result in a lower weathering effect if considering their effective interaction [32]. Phase diagrams were used here to pinpoint critical RH values for deliquescence, i.e., the threshold between crystallisation and dissolution of the above salt systems.

To estimate the biological risk in the Mithraeum, we assumed the microbiota frequently observed in Roman hypogeal sites. The Roman hypogeal site of the Catacombs of St. Callistus and Domitilla can be considered as one of the most extensive case studies on this topic [33] and can be reasonably used for similarity to the Mithraeum. In the Catacombs, it was found that actinobacteria and fungi—such as *Aspergillus*, *Cladosporium*, *Penicillium*, and *Sporotrichum*—are commonly detected with phototrophic microbes, as already generically mentioned by [20]. Among these heterotrophic biodeteriogens, mould fungi highly jeopardise mineral materials and can develop and grow even in the absence of a primary phototrophic biofilm, as long as nutrients are available on surfaces [21], e.g., organic compounds of pictorial layers or transported by air infiltration or visitors. In our study, the Sedlbauer isopleths were used to assess the risk of mould fungi colonisation both in terms of spore germination and mycelial growth [34]. The critical RH for mould germination and growth is associated to the Lowest Isopleth for Mould (LIM). The isopleths were drawn while assuming the worst case scenario. Substrate category I is compatible with the Mithraeum walls, which are non-biologically recyclable porous materials with considerable soiling.

### 2.4. The Risk Index

**Figure 2:**
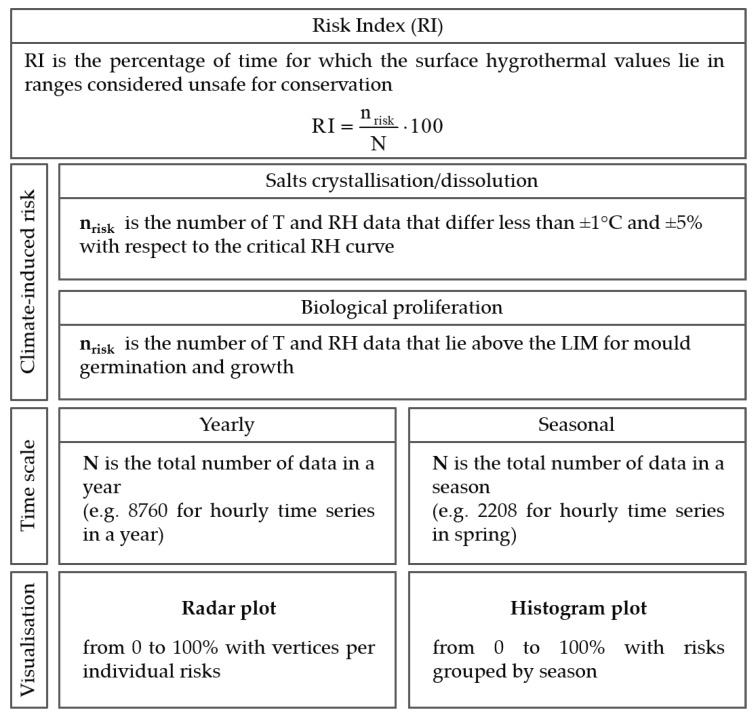
Schema of the Risk Index calculation and visualisation.

A Risk Index (RI) was introduced to synthesise the overall microclimate-induced crystallisation/dissolution and biological risks in hypogeal sites. In this context, the visualisation of T${}_{s}$-RH${}_{s}$ on scatter plots with critical RH curves is a powerful analytical tool. However, it requires detailed knowledge of the damage potential of each degradation agent and it can only be fully interpreted by expert users. In contrast, RI provides a fast and reliable diagnostic evaluation tool that can be used to more easily identify risks and assess their severity. RI is based on long-term hygrothermal time series measured in proximity to damaged surfaces, i.e., T${}_{s}$ and RH${}_{s}$, and it is defined as the percentage of time from 0 (no risk) to 100% (maximum risk) for which the surface hygrothermal values lie in ranges that are considered to be unsafe for conservation. A schema summarising the calculation of the Risk Index and the proposed final visualisation of the outputs is shown in Figure 2. For the crystallisation/dissolution risk, for each salt system, RI takes into account hygrothermal values differing less than ±1${}^{{}^{\circ}}$C and ±5% as compared to the critical RH curve. These conditions were chosen, as they might trigger harmful stress-and-strain cycles due to phase change of salts, which are the main factor responsible for the deterioration of porous materials. For the biological risk, RI is calculated when considering the hygrothermal values above LIM for mould germination, and growth, where proliferation can occur. Radar plots are used to visualise the individual risks on a yearly basis, while, in histogram plots, the risks are grouped by season. Both visualisation tools assist the comparison of the synergism among frequently occurring hazards inside hypogeal sites before and after maintenance interventions and over the course of years.

## 3. Results and Discussion

### 3.1. Temporal Behaviour of the Microclimate during the Year

**Figure 3:**
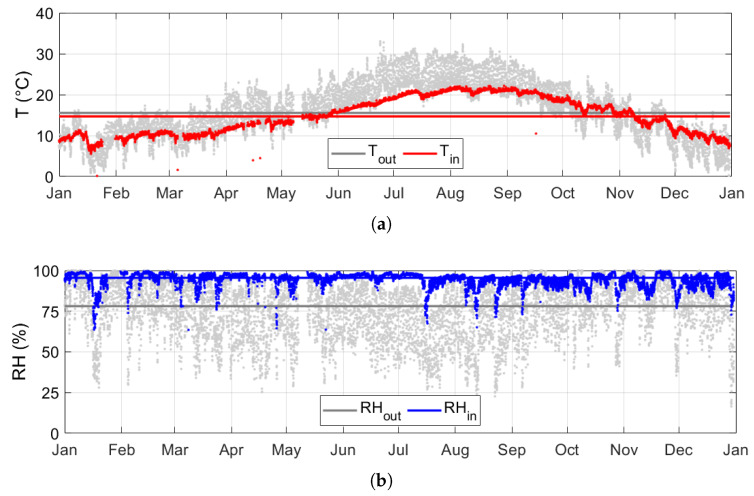
Hygrothermal conditions inside the Mithraeum in 2016: (**a**) temperature, (**b**) relative humidity.

The hygrothermal conditions in 2016 are reported in Figure 3 as being representative of the annual microclimate temporal behaviour inside the Mithraeum. This year was chosen, as it was associated to the most complete (CoI = 1) and continuous (CI = 1) time series that were collected among the years.

On a yearly basis, the indoor mean temperature significantly agreed with the outdoor one: T${}_{in}$ = 14.7 ${}^{{}^{\circ}}$C and T${}_{out}$ = 15.5 ${}^{{}^{\circ}}$C (Figure 3a). T${}_{in}$ was smoothed out with respect to T${}_{out}$ on both a seasonal and daily basis, respectively, showing a time-lag of one month in the T${}_{in}$ maximum peak due to conductive heat transfer through soil (high thermal inertia) and a time-lag of few days due to convective heat transfer. The RH values were close to saturation most of the year, ranging between 80% and 100% (Figure 3b). The median of RH${}_{in}$ was 95.5% and the median of RH${}_{out}$ = 78.1%, with drops in RH${}_{in}$ being related to simultaneous drops in RH${}_{out}$.

### 3.2. General Microclimate Analysis

The quality of T and RH time series were objectively evaluated before performing the comparison among microclimate data that were collected in three years: 2011 as representative of the microclimate conditions before the intervention, 2013 as representative of the period immediately following the intervention, and 2019 as representative of the most recent microclimate conditions. The preliminary investigation on the microclimate conditions inside the Mithraeum was limited to the time intervals available in all three years from 1st February until 30th April. In 2011, the environmental monitoring was temporarily suspended due to the starting of the maintenance intervention. For the selected periods, the CoI and CI values are close to unity, which means that the time series were both complete (few missing data) and continuous (few intervals with missing data), hence being suitable for studying the hygrothermal behaviour of the site.

**Figure 4:**
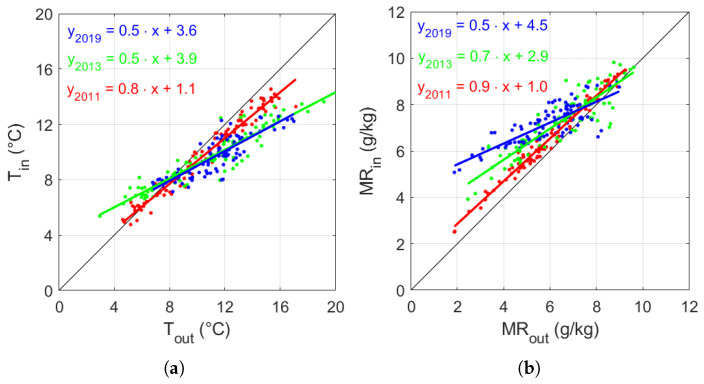
Scatter plot of daily mean indoor values *versus* outdoor values collected from 1st February until 30th April in 2011, 2013, and 2019: (**a**) air temperature (T) and (**b**) mixing ratio (MR). The linear regression equations for each dataset are reported together with the bisectrix (i.e., T${}_{in}$ = T${}_{out}$ and MR${}_{in}$ = MR${}_{out}$, in black).

The first comparison was carried out investigating the influence of the external weather on the indoor hygrothermal conditions. Looking at the linear regression equations reported on Figure 4a, the angular coefficient (*m*) of T in 2011 was close to unity, while *m* was halved in 2013 and 2019. Similarly, the value of *m* of the MR linear regressions (Figure 4b) dropped from 0.9 in 2011 to 0.5 in 2019, which might imply a progressive wetness of the site. This result can be interpreted as a direct consequence of the closure of the openings, as the indoor hygrothermal conditions tended to be less dependent on the outdoor values.

**Figure 5:**
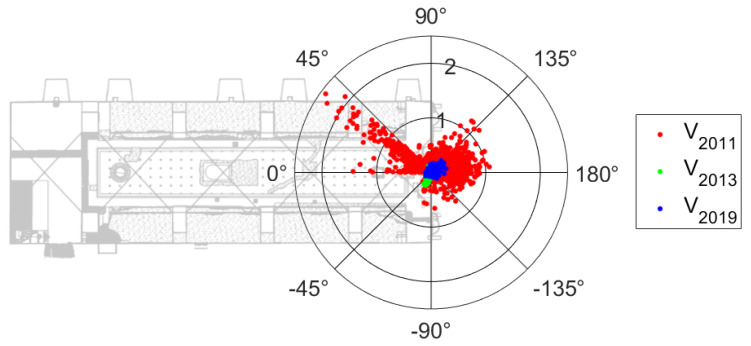
Polar plot of air flow intensity (V) and direction with respect to the major axis of the Mithraeum (on the right) and before (2011) and after the closure of the main openings (2013–2019).

The reduced variability found in T and MR in ambient air since 2013 (Figure 4) was confirmed by a substantial drop in the intensity of air currents (V) inside the Mithraeum (Figure 5). In 2011, the V values were well scattered, although a prevailing direction was noticeable, which ranged between 40${}^{{}^{\circ}}$ and 45${}^{{}^{\circ}}$, coming from one of the windows, which was also characterised by the V maximum of 2.3 m/s. In addition to the maintenance intervention, the hygrothermal conditions from October 2012 to January 2013 were affected by temporarily openings to visits. After the reduction of these forcing factors (i.e., natural ventilation and visitors), the dependency on the internal climate on the outdoor weather conditions was visibly reduced in the years 2013 and 2019, with data in the polar plot of air intensity less (2013–2019) scattered in the polar plot of V (Figure 5) and V always lower than 0.5 m/s.

The temporal behaviour of the hygrothermal conditions measured a few centimeters from the wall (sensor S${}_{wall}$ in Figure 1) was analysed while taking the observations of surface temperature (T${}_{s}$), air temperature (T), and mixing ratio (MR) values into account.

**Figure 6:**
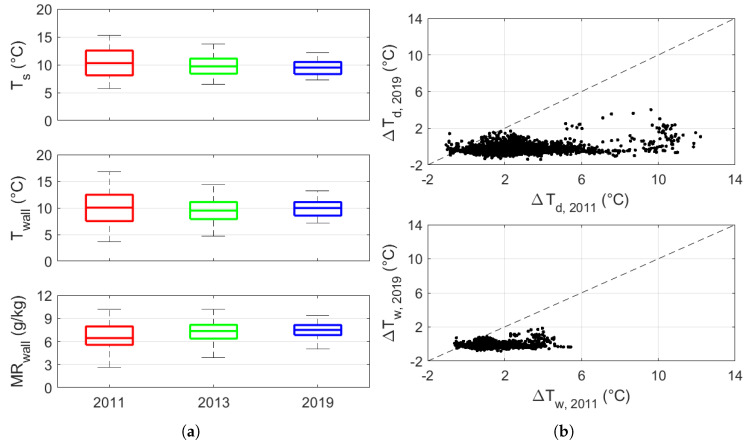
(**a**) Boxplots of wall surface temperature (T${}_{s}$), air temperature (T), and mixing ratio (MR), and (**b**) scatterplots of temperature difference between $T_{s}$ and $T_{d}$, i.e., ΔT${}_{d}$, and temperature difference between $T_{s}$ and $T_{w}$, i.e., ΔT${}_{w}$.

The box-and-whiskers plots of T${}_{s}$, T, and MR observations (Figure 6a) shows that the boxes fully overlapped over the years, meaning that no significant difference was detectable after the closure of the main openings. Indeed, the mean of the medians over the three years was 9.8 ${}^{{}^{\circ}}$C, ranging from 9.4 to 10.2 ${}^{{}^{\circ}}$C for T${}_{s}$, 9.7 ${}^{{}^{\circ}}$C ranging from 9.5 to 9.9 ${}^{{}^{\circ}}$C for T, and 6.9 g/kg ranging from 6.1 to 7.4 g/kg for MR. Conversely, the Interquartile Range (IQR, i.e., the difference between the third and first quartile) shows a decreasing trend year by year: from 10 to 5 ${}^{{}^{\circ}}$C for T${}_{s}$, from 13 to 6 ${}^{{}^{\circ}}$C for T, and from 8 to 4 g/kg for MR.

This effect is also evident in Figure 6b when comparing ΔT${}_{d}$ and ΔT${}_{w}$ before and after the retrofit. It is important to bear in mind that both of the parameters are based on isobaric cooling until saturation is reached, although T${}_{d}$ is related to cooling without changes in MR, whereas T${}_{w}$ is linked to cooling due to the evaporation of water. As the values collapsed to the condition where ΔT${}_{d}$ = ΔT${}_{dw}$ = 0 ${}^{{}^{\circ}}$C, the risk of condensation associated with both cooling processes greatly increased after the intervention.

**Figure 7:**
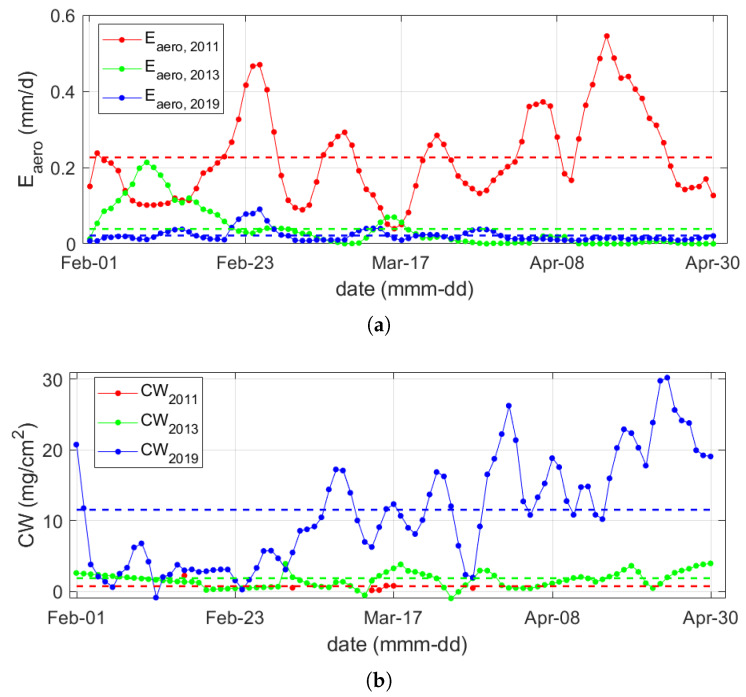
(**a**) Daily evaporation rate (E${}_{aero}$) and (**b**) daily condensed water (CW) before (2011) and after the intervention of maintenance (2013–2019). Average values are represented as dashed lines.

Two empirical functions were used to study the temporal behaviour of the daily evaporation rate (E${}_{aero}$, Equation (2)), and the amount of condensed water (CW, Equation (4)) on the walls. As shown in Figure 7a, in 2011 E${}_{aero}$ reached up to 0.74 mm/d, whereas in both 2013 and 2019 it was always below 0.10 mm/d, decreasing on average from 0.24 mm/d before the maintenance intervention to 0.02 mm/d after the maintenance intervention. Figure 7b shows that most of the occurrences of CW with values lower than 1.0 mg/cm${}^{2}$ occurred before the intervention (2011), whereas, in 2013 and 2019, CW was higher than 0.5 mg/cm${}^{2}$, reaching values up to 30.0 mg/cm${}^{2}$ in 2019. Moreover, the average CW tended to increase, at first, from 0.6 mg/cm${}^{2}$ in 2011 to 1.8 mg/cm${}^{2}$ in 2013, and then strongly to 11.1 mg/cm${}^{2}$ in 2019. Furthermore, in 2019, the CW values tended to raise over time, as P was always close to unity, whereas T${}_{s}$− T${}_{d}$ is close to zero.

### 3.3. Specific Risk Assessment

The conservation risk assessment was performed on the time series of the years 2011, 2013, and 2019, while taking the hygrothermal conditions in proximity to the walls into account (sensor S${}_{wall}$ in Figure 1). Mechanical and biological risk were investigated, as described in Section 2. It should be noticed that, in 2011, data were only collected from January to the end of April, as the maintenance intervention started in May.

**Figure 8:**
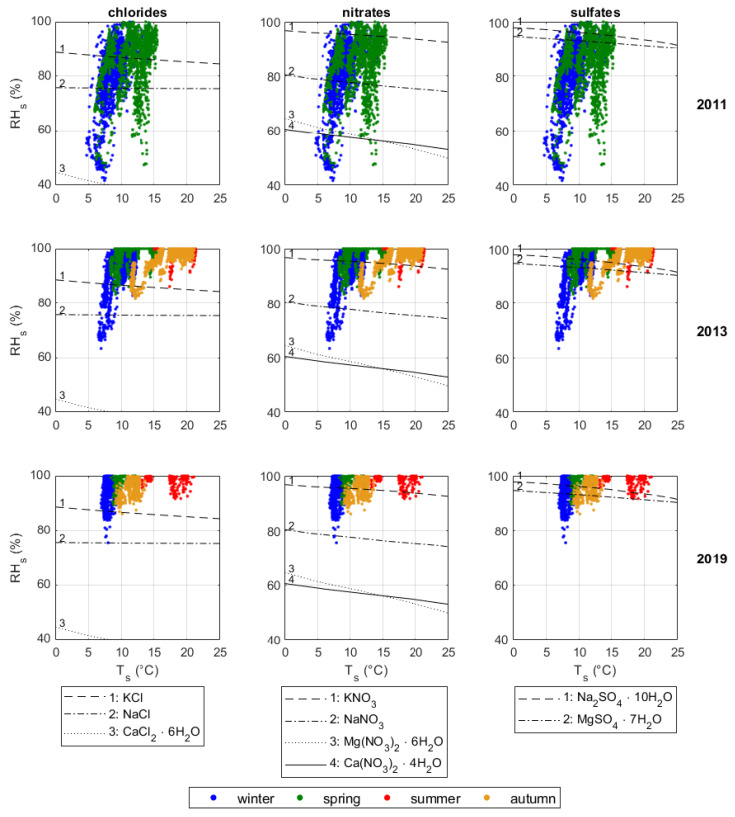
Relative humidity (RH${}_{s}$) *vs* temperature (T${}_{s}$) close to wall surfaces plotted on phase diagrams with the critical RH curve for deliquescence of chlorides, nitrates and sulfates. **Chlorides**: (1) sylvite (potassium chloride—KCl), (2) halite (sodium chloride—NaCl), (3) antarcticite (calcium chloride—CaCl_2 · 6(H_2O)). **Nitrates**: (1) nitre or nitrokalite (potassium nitrate—KNO_3), (2) nitratine (sodium nitrate—NaNO_3), (3) nitromagnesite (magnesium nitrate—Mg(NO_3)_2· 6(H_2O)), (4) nitrocalcite (calcium nitrate—Ca(NO_3)_2· 4(H_2O)). **Sulfates**: (1) mirabilite (sodium sulfate—Na_2SO_4 · 10(H_2O)), (2) epsomite (magnesium sulfate—MgSO_4 · 7(H_2O)).

The risk related to crystallisation was estimated by plotting T${}_{s}$ and RH${}_{s}$ on phase diagrams showing the critical RH for the deliquescence salts ordered by increasing harmfulness, i.e., chlorides, nitrates, and sulfates (Figure 8). In 2011 (first row of the 3 × 3 matrix), RH${}_{s}$ values spanned from 40% to saturation, which means that chlorides, nitrates, and sulfates could be present in both solid and dissolved forms. These conditions could be associated with high risk of crystallisation for all of the deliquescent salts considered, except for antarcticite. The risk associated with sodium chloride (halite) and potassium chloride (sylvite) could have produced negligible damage to the stone, limited to aesthetically-disturbing efflorescences on the surface [32]. Concerning mirabilite, the main conservation risk could be related to the formation of sub-efflorescence within the stone pores [35], threatening the conservation of porous materials because of its high crystallisation pressure [15].

After the intervention, the RH${}_{s}$ values always exceeded 60% due to the reduced ventilation in the Mithraeum. In 2013 (second row of the matrix), nitromagnesite and nitrocalcite could have only existed as dissolved ions, thus excluding the risk of mechanical damage caused by their precipitation. In 2019 (third row of the matrix), the RH${}_{s}$ values were always higher than 80% and only sylvite, nitrokalite, and the sulfates might have formed crystals. Nevertheless, the risk associated with the crystallisation of mirabilite might still remain high, as mechanical damage is governed by the frequency of the alternation between dissolution and precipitation.

**Figure 9:**
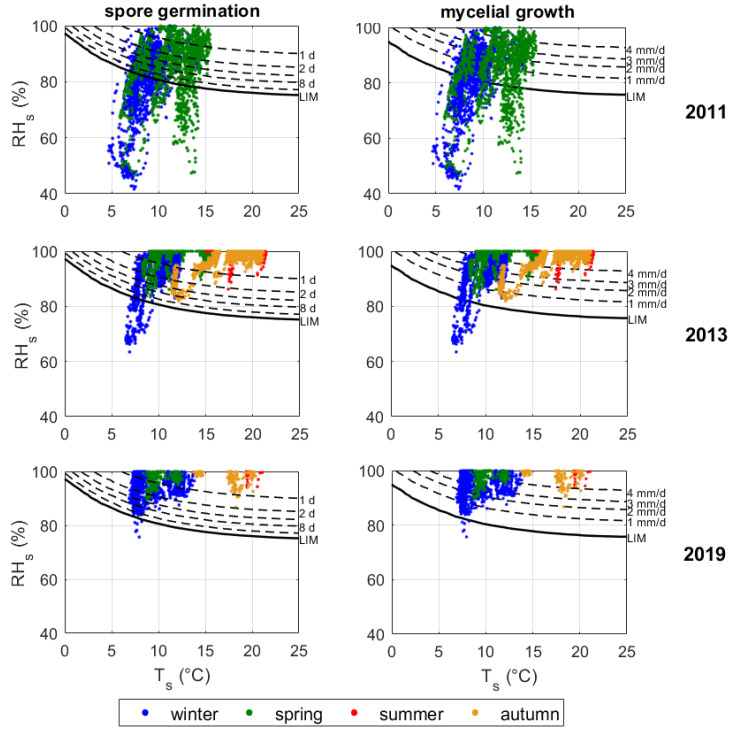
Relative humidity (RH${}_{s}$) *vs* temperature (T${}_{s}$) close to wall surfaces plotted on Sedlbauer curves (isopleths) for mould risk assessment on Substrate category I. Left panels: mycelial growth curves in mm/d; right panels: spore germination curves in days.

The risk of bio-colonisation by mould fungi was assessed by plotting the collected hygrothermal data on Sedlbauer isopleths describing spore germination (Figure 9, left panels) and mycelial growth (Figure 9, right panels) on substrates characterised by biologically adverse recyclable building materials with considerable soiling (Substrates category I). The biological risk greatly increased year by year, especially during winter and spring, as most data lied considerably beyond the LIM (Lowest Isopleth for Mould). Moreover, in summer and autumn of 2019 the risk of spore germination rose to eight days, whereas the mycelial growth is always higher than 1 mm/d if compared to data that were collected in 2013.

### 3.4. Application of the Risk Index

**Figure 10:**
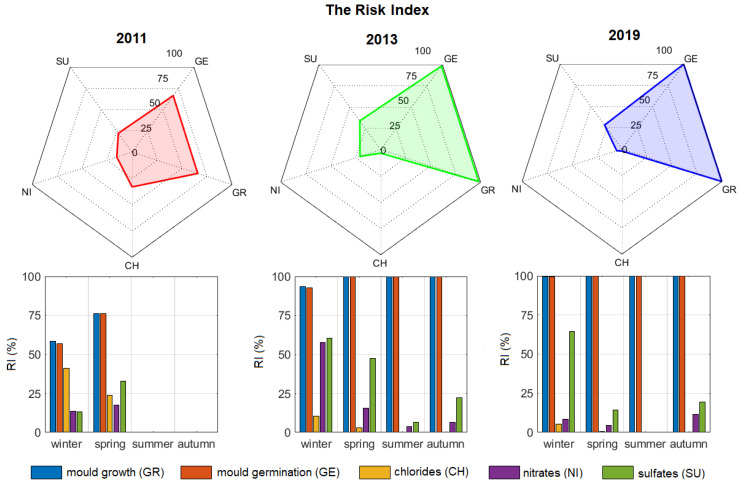
Comparison among Risk Index (RI) values calculated for individual risks before (2011) and after (2013-2019) the maintenance intervention. Upper panels: radar plot with RI (%) calculated on a yearly basis. Lower panels: histogram plots of RI (%) calculated on a seasonal basis.

The Risk Index (RI) for hypogeal sites was calculated on a yearly basis (Figure 10, upper panels) and in each season (Figure 10, lower panels) to synthetically characterise the biological proliferation and crystallisation/dissolution risks inside the Mithraeum.

In 2011 (Figure 10, left panels), the hygrothermal conditions promoted both the conservation risks, with RI ranging between 20% (nitrates) and 70% (germination and growth of spores) on a yearly basis. Deterioration seemed to be mainly governed by the air moisture content close to the wall surface. In spring, the increase in moisture content determined an enhanced biological risk and phase changes of sulfates, as well as an inhibition of phase changes of chlorides; on the contrary, nitrates did not seem to be affected by the moisture seasonal cycle. In 2013 and 2019, the biological risk significantly increased with respect to the condition in 2011 (RI = 100%), as the closure of the main openings limited air movements (Figure 5) and RH higher than 80% most of the time. On the contrary, the crystallisation risk diminished following a seasonal cycle where the most hazardous period was winter. In 2013 (Figure 10, mid panels), the risk of phase change of chlorides was reduced as compared to 2011 and limited to winter, when the site was still open to visits. Conversely, the risk of phase change of nitrates and sulfates increased, especially in winter and spring, showing a seasonal cycle with minimum in summer. This finding might be caused by the increased evaporation that occurred in winter 2013, as shown in Figure 7a. In this respect, crystallisation/dissolution risk seemed to be governed by temperature. In 2019 (Figure 10, right panels), the biological risk still remained high throughout the year (RI = 100%). As in 2013, the risk that was related to chlorides was low and limited to winter and spring. The risk of phase change of nitrates strongly decreased from 25% in 2013 to 5% in 2019, showing a low seasonal trend. The risk of crystallisation/dissolution alternation of sulfates was comparable with that in 2013, except for spring.

The visualisation of T${}_{s}$-RH${}_{s}$ on scatter plots with critical RH curves provided a meaningful risk assessment, which cannot be fully interpreted without detailed knowledge of the damage potential of individual degradation agents. In contrast, RI made a fast and reliable identification of the risks as well as an assessment of the severity levels possible. Indeed, the advantage of the application of RI was the ability to synthesise the study of long-term time series, providing a global characterisation of deterioration on both a yearly and a seasonal basis. In this perspective, RI might be used by heritage site managers as a practical parameter to effectively compare different individual risks throughout the years and after maintenance interventions. There is some uncertainty regarding the assumptions made in this study, as microclimate-induced deterioration in the Mithraeum could have been different from those most frequently occurring in other Roman hypogeal sites. However, the results can be generally applicable in the assessment of the conservation risks. The experimental verification of crystallisation/dissolution and bio-colonisation risks will be planned in future analytical campaigns to encompass the above limitation.

## 4. Conclusions

A comprehensive assessment of the microclimate-induced deterioration was performed inside the ”Mithraeum of the Baths of Caracalla” by evaluating the most common conservation risks in hypogeal sites, i.e., crystallisation/dissolution of deliquescent salts and mould fungi proliferation. The analysis of long-term microclimate series provided an accurate characterisation of the site over the years, clearly showing the effect of a maintenance intervention on the hygrothermal conditions. The investigation highlighted a reduced variability of temperature and relative humidity due to the limited internal air flows. The closure of the main openings has reduced the moisture exchanges with the outdoors, leading to an increase in the dampness conditions. The conservation risk close to wall surfaces was assessed on hygrothermal scatter plots with the critical RH curves of three deliquescent salts systems and of mould fungi proliferation. These diagrams provide an evaluation of the crystallisation and biological risks in response to the hygrothermal conditions. Such data visualisation cannot allow easy interpretation, especially in the case of crystallisation risk, as the mechanical deterioration is related not only to crystalline growth, but also to crystallisation and dissolution cycles of each saline solution around its critical RH curve.

The Risk Index (RI) provided a quantitative measure of the individual risks and made it possible to analyse their synergism, supporting the interpretation of diagrams of both deliquescent salts phase change and mould fungi proliferation. A synthetic visualisation of the overall risks was proposed by means of radar and histogram plots of RI, respectively, on a yearly and seasonal basis. The Risk Index proved to be effective in diagnosing and following up on the microclimate-induced risks in the hypogeal sites subjected to specific interventions. Although the Risk Index here was specifically designed for cultural hypogeal sites with long-term time series, RI can be adjusted to fit different aims and requirements. The evaluation of crystallisation/dissolution risk was limited to typical occurrences of deliquescent salts in Roman hypogeal sites, but it could be extended to other salt systems that are associated with different environmental conditions. The same applies to the biological risk, which focuses on spore germination and mould growth, but might also be conveniently extended to cyanobacteria and colonisation by insects.

Some final suggestions for improving the risk assessment procedure for future research perspectives are proposed. First, a database of microclimate-induced deterioration functions for cultural materials could be implemented in view of the application of RI to different environments and various conservation needs. Subsequently, a standalone executable of the RI algorithm can be developed to better support historical site managers and other stakeholders in tailoring preventive conservation strategies.

## Figures and Tables

**Table 1:** Studies dealing with indoor climate investigations within cultural and/or natural hypogeal sites listed per conservation risk assessment. CO${}_{2}$ = carbon dioxide concentration; E = irradiance; O${}_{2}$ = oxygen concentration; P = atmospheric pressure; RH = relative humidity; T = temperature; T${}_{s}$ = surface temperature; and, V = indoor wind intensity.

Year	Ref.	Site	Climate Variables	Conservation Risk Assessment
2005	[3]	catacombs	T, RH, CO${}_{2}$, P	biological risk
2014	[4]	caves	T, RH, CO${}_{2}$	
2016	[5]	mithraeum	T, RH, E, CO${}_{2}$	
2014	[6]	catacombs	T, RH, E, CO${}_{2}$, V	biological and crystallisation risks
2004	[7]	archaeological site	T, RH, V	crystallisation risk
2011	[8]	necropolis	T, RH	
2014	[9]	crypt	T, RH, T${}_{s}$, V	
2008	[10]	tombs ${}^{\left(*\right)}$	T, RH, V	air flow patterns due to visitors
2019	[11]	burial sites ${}^{\left(**\right)}$	T, RH, T${}_{s}$, V, CO${}_{2}$	
2010	[12]	cave settlements ${}^{\left(**\right)}$	T, RH	energy and indoor climate performance
2012	[13]	archaeological site	T, RH, E, CO${}_{2}$, O${}_{2}$	–
2018	[14]	mithraeum and tomb	T, RH, V	

${}^{\left(*\right)}$ CFD simulation; ${}^{\left(**\right)}$ on-site measurements and CFD simulation.

## Abbreviations

The following abbreviations are used in this manuscript. Derived parameters are marked in italics.

CW	Condensed Water
E${}_{aero}$	Evaporation rate
LIM	Lowest Isopleth for Mould
MR	Mixing Ratio
RH	Relative Humidity
RH${}_{s}$	surface Relative Humidity
T	Temperature
T${}_{max}$	daily maximum Temperature
P	time of Permanence below T${}_{d}$
T${}_{d}$	dew point Temperature
T${}_{s}$	surface Temperature
T${}_{w}$	wet bulb Temperature
V	air flow intensity
*Greek abbreviations*	
$\Delta T_{d}$	temperature difference between T${}_{s}$ and T${}_{d}$
$\Delta T_{w}$	temperature difference between T${}_{s}$ and T${}_{w}$
